# Recombinant Human C1 Esterase Inhibitor for the Management of Adverse Events Related to Intravenous Immunoglobulin Infusion in Patients With Common Variable Immunodeficiency or Polyneuropathy: A Pilot Open-Label Study

**DOI:** 10.3389/fimmu.2021.632744

**Published:** 2021-03-02

**Authors:** Isaac R. Melamed, Holly Miranda, Melinda Heffron, Joseph R. Harper

**Affiliations:** ^1^ IMMUNOe Research Centers, Centennial, CO, United States; ^2^ Pharming Healthcare Inc., Warren, NJ, United States

**Keywords:** angioedemas hereditary, complement C1 inhibitor protein, immunoglobulins intravenous, recombinant human C1 esterase inhibitor, Ruconest

## Abstract

It has been hypothesized that low levels of C1 esterase inhibitor (C1-INH), a key inhibitor of the complement pathway, may play a role in the occurrence of adverse events (AEs) associated with intravenous immunoglobulin (IVIG) therapy. This open-label pilot study evaluated C1-INH replacement, with recombinant human C1-INH (rhC1-INH), as a potential therapy for adults requiring IVIG and experiencing AEs. Patients received two rounds of IVIG infusion [pre-treatment phase (no rhC1-INH), 4–8 weeks] and then three rounds of one dose of intravenous rhC1-INH 50 U/kg (maximum, 4,200 U) with subsequent IVIG infusion (treatment phase, 6–12 weeks). Nineteen adults completed the study; all had an autoimmune condition linked to common variable immunodeficiency (CVID) or polyneuropathy, and 57.9% had low baseline C1-INH levels. Mean ± SD total scores improved significantly with the Headache Impact Test (from 62.8 ± 6.2 at pre-treatment to 57.7 ± 9.1 after treatment; mean Δ, −5.0; *p* = 0.02) and Modified Fatigue Impact Scale (from 59.3 ± 13.1 to 51.2 ± 15.4; mean Δ, −8.1; *p* = 0.006). Significant improvements in the Migraine Disability Assessment were observed for three of five items (*p* ≤ 0.002). Mean ± SD C1-INH level increased from 26.8 ± 5.9 mg/dl after the second round of IVIG (pre-treatment) to 32.1 ± 7.8 mg/dl after the third rhC1-INH treatment; functional C1-INH levels increased from 115.8 ± 34.7% to 158.3 ± 46.8%. Future research is warranted to explore the benefit of C1-INH therapy for reduction of IVIG-related AEs, as well as the role of C1-INH in patients with CVID and autoimmune disease.

**Clinical Trial Registration:**

ClinicalTrials.gov, identifier NCT03576469.

## Introduction

Intravenous immunoglobulin (IVIG) is administered to treat a variety of immunodeficiency, autoimmune, and inflammatory conditions (1, 2). Common variable immunodeficiency (CVID) is a form of severe antibody deficiency that is characterized by infections, gastrointestinal disorders, autoimmune diseases, and increased susceptibility to malignancies (3, 4). Replacement therapy with IVIG reduces infection rates in patients with various forms of primary immunodeficiency, including CVID (1, 5). Although generally considered safe, IVIG therapy is associated with adverse events (AEs), notably headache and fatigue (1, 4–7).

The mechanisms underlying IVIG-related AEs have yet to be fully elucidated. One possible mechanism is activation of complement resulting from an interaction between infused antibodies and antigens present in the patient (*eg*, infectious agents) or the presence of prekallikrein activators or kallikrein in IVIG preparations (1, 7). Exploratory data suggest that levels of C1 esterase inhibitor (C1-INH), a key inhibitor of the complement pathway, may be linked to CVID (8). A subgroup of patients with CVID who commonly experienced IVIG-related AEs were found to have lower than normal C1-INH and/or functional C1-INH levels (8). There was an apparent downregulation of C1-INH levels after IVIG therapy and an associated increase in the incidence of IVIG-related AEs, suggesting a possible relationship (8). Additionally, further findings suggest low level and function of C1-INH may play a role in the relationship between a post-infectious response and neurologic changes, a form of post-infectious autoimmunity that results in various neurologic symptoms (9).

Recombinant human C1-INH (rhC1-INH; Ruconest) is approved in numerous countries for the treatment of acute attacks in adolescents and adults with hereditary angioedema (HAE) (10). In HAE, reduced serum levels or a functional deficiency of the C1-INH protein results in uncontrolled activation of the contact enzyme systems and leads to overproduction of bradykinin, which causes angioedema of subcutaneous and/or submucosal tissues (11, 12). Replacement therapy with rhC1-INH increases plasma levels of functional C1-INH (13) and resolves angioedema symptoms in patients with HAE (14–19). Given the hypothesis that low levels of C1-INH may play a role in the incidence of IVIG-related AEs, the current pilot study was conducted to evaluate C1-INH replacement therapy, using rhC1-INH, as a potential therapy for adults who require IVIG and have experienced IVIG-related AEs.

## Materials and Methods

### Study Design and Participants

This single-center, investigator-initiated, open-label pilot study was conducted from June 28, 2018 to July 12, 2019 (ClinicalTrials.gov identifier: NCT03576469). Adults aged ≥18 years who were receiving a stable dose of IVIG therapy (≥ three infusions) and experienced IVIG-related AEs after a routine infusion were eligible for inclusion. Exclusion criteria included receiving treatment for HAE (prophylactic or acute therapy) and history of allergy to rabbits or rabbit-derived products. The study was conducted in accordance with the Good Clinical Practice guidelines of the International Conference on Harmonization and applicable local regulatory requirements. The study protocol was approved by an independent review board (IntegReview IRB, Austin, TX), and all patients provided written informed consent.

**Figure 1:**
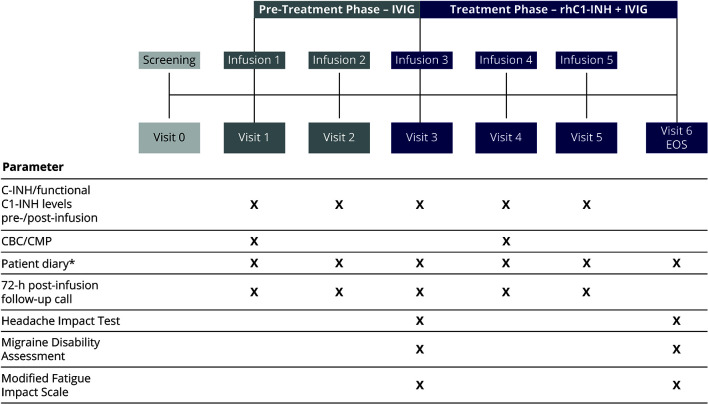
Study design. ^*^For recording AEs, concomitant medications, infections, hospitalizations, physician and/or emergency room visits, and school and/or work days missed because of infection or illness. AEs, adverse events; C1-INH, C1 esterase inhibitor; CBC, complete blood count; CMP, comprehensive metabolic panel; EOS, end of study; IVIG, intravenous immunoglobulin; rhC1-INH, recombinant human C1 esterase inhibitor.

The study consisted of a screening visit, a pre-treatment phase of 4–8 weeks, and a treatment phase of 6–12 weeks (**Figure 1**). During the pre-treatment phase, eligible patients received two rounds of their previous IVIG infusion. The treatment phase with C1-INH replacement therapy consisted of three rounds of intravenous rhC1-INH, with each rhC1-INH infusion immediately followed by an infusion of IVIG. During each round of the treatment phase, the rhC1-INH dose was determined based on the patient’s body weight; rhC1-INH was then administered as a single intravenous dose of 50 U/kg (maximum dose, 4,200 U). This dose of rhC1-INH has been shown to achieve levels above the lower limit of normal in patients with C1-INH deficiency (*eg*, HAE) (13). IVIG infusions were administered according to the product package insert, including adjustment of the infusion rate based on tolerability as recommended by the manufacturer.

### Assessments

Patient diaries were used to record AEs through 72 h after each infusion of IVIG, in both the pre-treatment and treatment phases (**Figure 1**). In addition, self-reported measures of IVIG-related AE severity were administered before the third infusion (to evaluate the pre-treatment phase) and at the end of study visit (to evaluate the treatment phase). The Headache Impact Test (HIT) consists of six items rated from “never” to “always” (score range, 36–78), with higher scores indicating a greater negative impact (20). The Migraine Disability Assessment (MIDAS) evaluates the impact of all headaches (not only migraines); in the current study, respondents reported the number of days after each infusion that headaches interfered with various activities (composed of five items) (21). The Modified Fatigue Impact Scale (MFIS) consists of 21 items rated from 0 (“never”) to 4 (“almost always”); total score range is 0–84, with higher scores indicating a greater negative impact (22). In addition to the total score, the MFIS yields three subscale scores: physical (score range, 0–36), cognitive (score range, 0–40), and psychosocial (score range, 0–8).

Blood samples were collected pre-infusion and 10 min after7nbsp;IVIG infusion (pre-treatment phase) or rhC1-INH + IVIG infusions (treatment phase). Total serum C1-INH concentration and functional C1-INH level were assessed prospectively during the study using standardized procedures at a single laboratory (National Jewish Health^®^ Advanced Diagnostic Laboratories, Denver, CO).

### Statistical Analysis

Changes from pre-treatment to posttreatment in HIT, MIDAS, and MFIS scores were analyzed using a t-test or analysis of variance (if data were normally distributed) or Wilcoxon rank-sum tests (if data were not normally distributed). Statistical tests were two-sided, with an alpha level of 0.05. Total serum C1-INH concentration and functional C1-INH level were summarized using descriptive statistics. Spearman’s correlation coefficient was used to evaluate the association between C1-INH (serum and functional) levels and IVIG-related AE assessment scores.

## Results

Twenty-six patients were screened; four patients did not meet eligibility criteria, and three enrolled patients withdrew consent. Overall, 19 adults completed the study and were receiving IVIG infusions (Octagam 10%, n = 5; Octagam 5%, n = 4; Gammagard, n = 4; Gammaked, n = 3; Gammaplex, n = 2; Gammunex, n = 1) as treatment for CVID (84.2%) or polyneuropathy (15.8%; **Table 1**). Based on previous testing, serum C1-INH concentration was low (≤20 mg/dl) in 57.9% of patients and functional C1-INH level was low (<67%) in 31.6% of patients (**Table 1**).

**Table 1:** Patient demographics and baseline characteristics.

Parameter	Patients (*N* = 19)
Age, years	
Mean (SD)	46.9 (13.0)
Range	21–63
Female, n (%)	18 (94.4)
Race, n (%)	
White	19 (100.0)
Diagnosis, n (%)	
Common variable immunodeficiency	16 (84.2)
Polyneuropathy	3 (15.8)
Complement deficiency, n (%)^*^	
Low serum C1-INH level	11 (57.9)
Low functional C1-INH level	6 (31.6)
Comorbidities, n (%)^†^	
Atopy	17 (89.5)
Autoimmune thyroiditis	16 (84.2)
GI disturbances	15 (78.9)
Arthralgia	14 (73.7)
Chronic fatigue syndrome	12 (63.2)
Cognitive impairment	12 (63.2)
Neuropathy	12 (63.2)

^*^Based on historical laboratory data (LabCorp, Englewood, CO) for each patient; low levels defined as serum C1-INH ≤20 mg/dl or functional C1-INH <67%.

^†^>50% of patients.

C1-INH, C1 esterase inhibitor; GI, gastrointestinal; SD, standard deviation.

**Figure 2:**
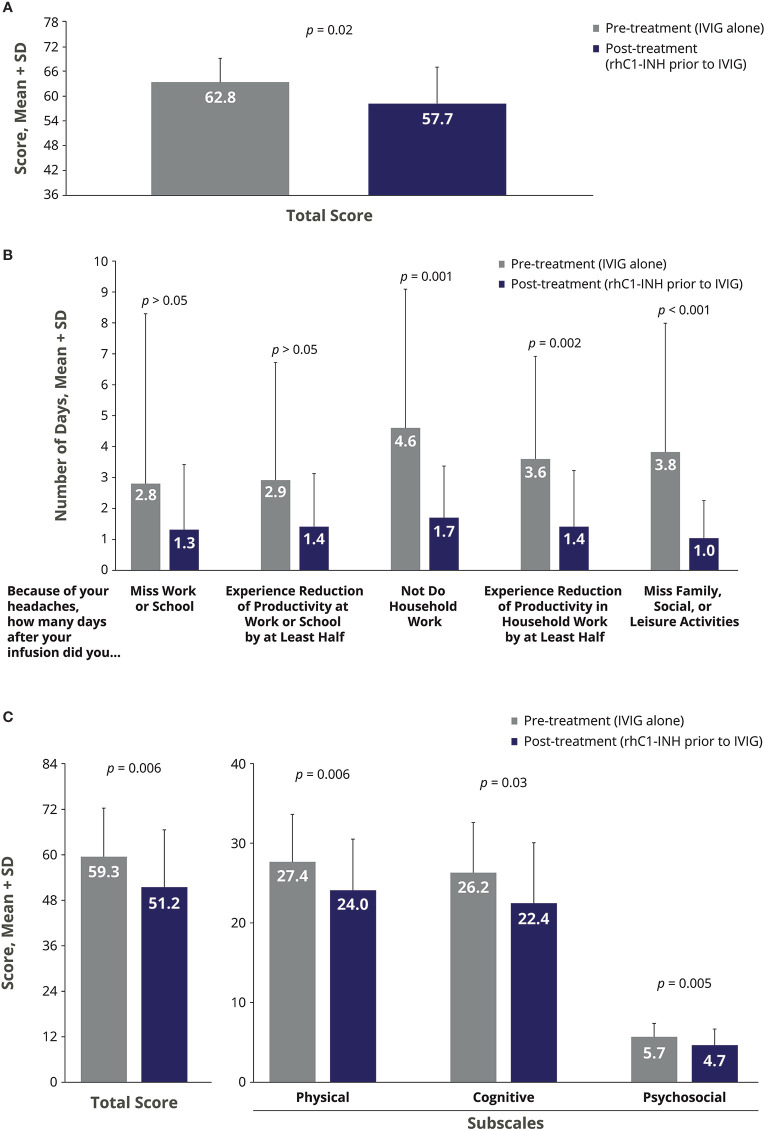
Impact of rhC1-INH prior to IVIG administration on AE severity in IVIG-treated patients as assessed by the **(A)** Headache Impact Test, **(B)** Migraine Disability Assessment, and **(C)** Modified Fatigue Impact Scale. Potential score ranges for the Modified Fatigue Impact Scale are 0–84 for total score, 0–36 for physical subscale, 0–40 for cognitive subscale, and 0–8 for psychosocial subscale. AE, adverse event; IVIG, intravenous immunoglobulin; rhC1-INH, recombinant human C1 esterase inhibitor; SD, standard deviation.

There were significant improvements in the severity of IVIG-related AEs during the treatment phase of rhC1-INH + IVIG compared with the pre-treatment phase of IVIG alone (**Figures 2A–C**). For HIT, the mean change with rhC1-INH + IVIG *vs.* IVIG alone was −5.0, indicating significant improvement (*p* = 0.02; **Figure 2A**). For MIDAS, a significant mean decrease (improvement) with rhC1-INH + IVIG *vs.* IVIG alone was observed in the number of days that headaches prevented household work (mean Δ, −2.7 days; *p* = 0.001); resulted in reduced productivity in household work by at least half (mean Δ, −2.1 days; *p* = 0.002); and interfered with family, social, or leisure activities (mean Δ, −2.7 days; *p* < 0.001; **Figure 2B**). There was also a decrease in the number of days that headaches caused a work or school absence (mean Δ, −1.2 days) and in the number of days that headaches caused a loss of productivity of at least half at work or school (mean Δ, −1.2 days), but differences *vs.* IVIG alone were not statistically significant (**Figure 2B**). For MFIS, statistically significant improvements *vs.* IVIG alone were observed for the total score (mean Δ, −8.1) and the physical (mean Δ, −3.4), cognitive (mean Δ, −3.7), and psychosocial (mean Δ, −1.0) subscale scores (**Figure 2C**). No serious AEs were reported.

The mean ± SD serum C1-INH level increased from 26.8 ± 5.9 mg/dl at Visit 2 (IVIG alone; no rhC1-INH) to 32.1 ± 7.8 mg/dl at Visit 5 (rhC1-INH prior to IVIG). In addition, the mean ± SD functional C1-INH level increased from 115.8 ± 34.7% to 158.3 ± 46.8%. Serum and functional C1-INH levels negatively correlated with HIT total score and MIDAS item scores when evaluated during the pre-treatment phase (IVIG alone) and during the rhC1-INH + IVIG treatment phase (**Table 2**). There was moderate correlation between C1-INH levels and HIT total scores (pre-treatment [IVIG alone]) and MIDAS productivity items scores (rhC1-INH + IVIG treatment phase).

**Table 2:** Correlation between serum C1-INH and functional C1-INH levels and IVIG-related AE assessment scores.

IVIG-Related AE Assessment	Spearman’s Correlation Coefficient^*^			
	Serum C1-INH Level		Functional C1-INH Level	
	Pre-Treatment (IVIG Alone)	Post-Treatment (rhC1-INH Prior to IVIG)	Pre-Treatment (IVIG Alone)	Post-Treatment (rhC1-INH Prior to IVIG)
Headache Impact Test				
Total score	−0.57	−0.26	**−0.50**	−0.18
Migraine Disability Assessment				
Miss work or school	−0.18	−0.18	−0.12	−0.12
Productivity at work or school reduced by at least half	−0.13	**−0.45**	−0.21	−0.30
Not do household work	−0.24	−0.38	−0.23	−0.26
Productivity in household work reduced by at least half	−0.14	**−0.50**	−0.07	**−0.45**
Miss family, social, or leisure activities	−0.26	−0.11	−0.33	−0.16
Modified Fatigue Impact Scale				
Total score	0.00	−0.09	−0.07	−0.02
Physical subscale score	0.05	−0.16	−0.03	−0.09
Cognitive subscale score	0.13	−0.07	0.04	0.04
Psychosocial subscale score	−0.10	−0.01	−0.15	−0.03

^*^Bold text represents moderate correlation.

AE, adverse event; C1-INH, human C1 esterase inhibitor; IVIG, intravenous immunoglobulin; rhC1-INH, recombinant human C1 esterase inhibitor.

## Discussion

Results from this pilot study support the hypothesis that low levels of C1-INH may play a role in the severity of IVIG-related AEs. Low serum C1-INH levels were documented in approximately 60% of the 19 patients included in the study. In this population of patients experiencing AEs after IVIG infusions, C1-INH replacement therapy with rhC1-INH increased serum C1-INH concentrations and was associated with significant reductions in the extent to which headaches interfered with daily activities and in the detrimental effects of fatigue.

In patients with CVID, IVIG infusions are typically administered every 3–4 weeks (4, 5). Adverse reactions to IVIG therapy may be acute (*eg*, headache, nausea) or may occur up to 72 h after infusion (*eg*, headache, fatigue) (4). These AEs have been linked to treatment-specific factors (*eg*, rapid infusion rates, change in IVIG product, delay since previous infusion) and patient characteristics (*eg*, concurrent or recent infection, no previous IVIG treatment) (23, 24). It has been proposed that low levels of C1-INH or functional C1-INH may also predispose patients to IVIG-related AEs (8), and that infusion of IVIG drives consumption of complement proteins, further lowering C1-INH levels and promoting inflammatory reactions (8). A relationship has been observed between the concentration of the solution (*ie*, protein density) and IVIG-related AEs (25). It is possible that a high rate of infusion of high-density proteins may activate HAE-like events in patients with low or borderline-low C1-INH levels.

One hypothesis is that high-density proteins downregulate C1-INH by increasing consumption of complement proteins, thereby causing headache in mild or moderate cases and aggravating the HAE-like event to “aseptic meningitis” in severe cases. The presentation of headache as a symptom in patients with HAE (26–28) as well as those receiving IVIG (25, 29) and the successful treatment of HAE-associated headaches with C1-INH (26) support this potential mechanism. Furthermore, a reduction in IVIG-related headaches may be obtained by providing IV hydration before and after IVIG therapy (6), supporting the hypothesis about the impact of protein density on C1-INH regulation. For patients who experience AEs with an IVIG 10% formulation, switching to an IVIG 5% formulation or switching to subcutaneous administration may help minimize AE occurrences or severity (25). It is possible that subcutaneous delivery does not interact with the complement system to downregulate C1-INH, which may explain the observed reduction in AEs relative to IV infusion; however, research is needed to evaluate this hypothesis. Addition of C1-INH replacement therapy is another potential option for addressing IVIG-related AEs, particularly in patients for whom C1-INH or functional C1-INH levels are close to or below the lower limit of normal. Because patients with CVID and low serum C1-INH levels may also exhibit edema (*eg*, limbs, face, abdomen) (8), rhC1-INH therapy, which is approved for the acute treatment of HAE attacks, may help minimize or resolve these symptoms as well.

Study limitations include the exploratory nature of the study, the small sample size, the lack of a control group, open-label study design, and assumptions related to the timing (immediately prior to IVIG administration) and appropriate dosing (50 U/kg body weight; maximum, 4,200 U) of rhC1-INH. Despite the small sample size and associated lack of statistical power, statistically significant decreases were observed in measures of headache and fatigue severity. Whether these changes are clinically meaningful still needs to be determined because there are currently no thresholds in minimally clinically important differences established for the outcomes measured (*ie*, HIT, MIDAS, and MFIS) in patients with CVID who are receiving IVIG therapy. In this study, rhC1-INH was administered before IVIG infusion at the approved dose for the treatment of HAE. It is possible that higher doses of rhC1-INH and/or administration both before and after IVIG infusion may provide more robust effects. These changes in rhC1-INH dosing and administration warrant further study in patients with IVIG-associated AEs.

## Conclusion

Administration of rhC1-INH prior to IVIG infusion may help ameliorate the severity of IVIG-related AEs. Future research is warranted to explore the benefit of C1-INH therapy in reduction of IVIG-related AEs, as well as the role of C1-INH in patients with CVID and autoimmunity.

## Data Availability Statement

The original contributions presented in the study are included in the article material. Further inquiries can be directed to the corresponding author.

## Ethics Statement

This study, which involved human participants, was conducted in accordance with the Good Clinical Practice guidelines of the International Conference on Harmonization and applicable local regulatory requirements. The study protocol was approved by an independent review board (IntegReview IRB, Austin, TX), and all patients provided written informed consent to participate in this study.

## Author Contributions

IRM and MH contributed to the study design and to the collection, analysis, and interpretation of the data. HM contributed to the collection, analysis, and interpretation of the data. JRH contributed to the analysis and interpretation of the data. All authors contributed to the article and approved the submitted version.

## Funding 

Funding for this investigator-initiated pilot study was provided by Pharming Healthcare Inc. Pharming Healthcare Inc. provided recombinant human C1 esterase inhibitor and funding for statistical analyses, as well as interpreted the data.

## Conflict of Interest

IRM reports serving as a speaker and an advisory board member for Pharming Group NV. JRH is an employee of Pharming Healthcare Inc.

The remaining authors declare that the research was conducted in the absence of any commercial or financial relationships that could be construed as a potential conflict of interest.

The authors declare that this study received funding from Pharming Healthcare Inc. The funder had the following involvement in the study: interpretation of data and medical accuracy review of the article content.
